# Enhancing the Cellular Uptake and Antibacterial Activity of Rifampicin through Encapsulation in Mesoporous Silica Nanoparticles

**DOI:** 10.3390/nano10040815

**Published:** 2020-04-24

**Authors:** Paul Joyce, Hanna Ulmefors, Sajedeh Maghrebi, Santhni Subramaniam, Anthony Wignall, Silver Jõemetsa, Fredrik Höök, Clive A. Prestidge

**Affiliations:** 1 Department of Physics, Chalmers University of Technology, SE-412 96 Gothenburg, Sweden; paul.joyce@unisa.edu.au (P.J.); silver@chalmers.se (S.J.); fredrik.hook@chalmers.se (F.H.); 2 School of Pharmacy & Medical Sciences, University of South Australia, Adelaide, South Australia 5090, Australia; hanna.gustafsson@chalmers.se (H.U.); sajedehsadat.maghrebi@mymail.unisa.edu.au (S.M.); santhni.subramaniam@mymail.unisa.edu.au (S.S.); anthony.wignall@unisa.edu.au (A.W.); 3 ARC Centre of Excellence in Bio-Nano Science and Technology, University of South Australia, Adelaide, South Australia 5090, Australia

**Keywords:** mesoporous silica, nanoparticle, permeability, antibiotics, total internal reflection, fluorescence microscopy, Caco-2, infection, small colony variants, *Staphylococcus aureus*

## Abstract

An urgent demand exists for the development of novel delivery systems that efficiently transport antibacterial agents across cellular membranes for the eradication of intracellular pathogens. In this study, the clinically relevant poorly water-soluble antibiotic, rifampicin, was confined within mesoporous silica nanoparticles (MSN) to investigate their ability to serve as an efficacious nanocarrier system against small colony variants of *Staphylococcus aureus* (SCV *S. aureus*) hosted within Caco-2 cells. The surface chemistry and particle size of MSN were varied through modifications during synthesis, where 40 nm particles with high silanol group densities promoted enhanced cellular uptake. Extensive biophysical analysis was performed, using quartz crystal microbalance with dissipation (QCM-D) and total internal reflection fluorescence (TIRF) microscopy, to elucidate the mechanism of MSN adsorption onto semi-native supported lipid bilayers (snSLB) and, thus, uncover potential cellular uptake mechanisms of MSN into Caco-2 cells. Such studies revealed that MSN with reduced silanol group densities were prone to greater particle aggregation on snSLB, which was expected to restrict endocytosis. MSN adsorption and uptake into Caco-2 cells correlated well with antibacterial efficacy against SCV *S. aureus*, with 40 nm hydrophilic particles triggering a ~2.5-log greater reduction in colony forming units, compared to the pure rifampicin. Thus, this study provides evidence for the potential to design silica nanocarrier systems with controlled surface chemistries that can be used to re-sensitise intracellular bacteria to antibiotics by delivering them to the site of infection.

## 1. Introduction

Harmful pathogens, such as *Staphylococcus aureus*, have evolved a multitude of evasive and defensive mechanisms that promote their survival against conventional antimicrobial agents—one of which is their ability to be internalized within host cells, protecting them from immune responses and conventional antibiotics [1,2]. Subsequently, intracellular pathogens are the source of several infectious diseases with limited treatment options and, thus, remain a major pharmaceutical challenge [3,4]. In order to treat pathogens residing in the intracellular environment, antibacterial agents must be delivered to the site of infection. Conventional antibiotics, such as rifampicin, suffer from low solubilities and/or low permeabilities, which restricts their ability to diffuse or be actively transported across the cellular membrane [5]. Subsequently, it has been estimated that over two-thirds of prescribed antibiotics are ineffective against intracellular pathogens [6]. Thus, a growing urgency exists for the development of new classes of antibiotics capable of being efficiently internalized by infected cells.

Intracellular pathogens can reside in an array of host cells, including immune cells, epithelial cells and bone cells [7,8,9]. Of increasing complexity with respect to treatment approaches are intracellular infections that reside within epithelial cells of the gastrointestinal (GI) tract, since physiological processing throughout the GI tract presents new obstacles for drug delivery [10]. For example, a wide range of antimicrobial peptides and proteins have been developed in recent years that exhibit bactericidal activities by disrupting bacterial cell membranes and have subsequently demonstrated in vitro efficacies in the treatment of intracellular pathogens [11,12]. However, while such biopolymers present promise for clinical translation in the treatment of some intracellular infections, their application to bacteria residing in the GI tract is limited due to stability issues, specifically within the harsh enzymatic and acidic conditions of the gastric environment [13].

An alternative delivery approach with demonstrated efficacy [14] and potential application for treating intracellular infections residing in the GI tract is to increase the antibacterial activity of conventional antibiotics by encapsulating them within nanocarriers that readily undergo endocytosis, thus delivering the antibiotic to the localized site of infection [15,16]. For this to serve as an effective approach, the nanocarrier system must: (i) have high physicochemical stability within the GI tract, (ii) protect the antibiotic from burst release prior to cell uptake, (iii) be effectively internalized within the target cell/s, (iv) release the drug within the intracellular compartments, ideally localizing drug release to the site of infection, and (v) be non-toxic to all host cells [17].

One such biocompatible nanocarrier system that has been used extensively to deliver drugs intracellularly, including within intestinal epithelium cells [18,19,20], and has been widely used for oral delivery, is that of mesoporous silica nanoparticles (MSN) [21,22]. MSN offer a number of delivery advantages, including their high physicochemical stability, nanoscale size which promotes rapid endocytosis by host cells, and ability to confine drugs with varying polarities at high concentrations [23,24]. Subramaniam et al. [14] recently investigated the ability for MSN, with varying particle sizes, to be internalized within macrophages for the treatment of small colony variants of *S. aureus* (SCV *S. aureus*). It was established that MSN enhanced the localized rifampicin concentration within the infected macrophages, which significantly reduced the number of live bacteria present within the cells and, thus, increased the bactericidal activity of rifampicin. However, macrophages have distinct uptake mechanisms that promote internalization of foreign bodies, which vary considerably to epithelial cells. Thus, it is unclear whether this formulation approach will serve as an effective treatment for eradicating bacteria shielded within epithelial cells, such as Caco-2 cells.

In this study, we investigate the ability for MSN particles, specifically with varying to surface chemistries and two different particle sizes, to be internalized by Caco-2 cells infected with SCV *S. aureus*, which serves as a model system simulating infected intestinal epithelial cells [25]. Hiroshima mesoporous material (HMM)-type MSN were selected and synthesized for this study, since the synthesis approach allows for spherical particles with controllable diameters < 100 nm and pore sizes > 5 nm, thus promoting efficient cell uptake and sufficient drug loading, respectively, when compared with other conventional MSN types [26,27,28]. By adopting facile changes to the synthesis approach, three sets of nanoparticles were developed with varying surface chemistries and particle sizes to allow for elucidating their role on cellular uptake and antibacterial activity of the encapsulated antibiotic, rifampicin. The impact of surface chemistry and particle size on the biophysical interaction with lipid membranes was further investigated using advanced surface-sensitive characterization techniques, specifically quartz crystal microbalance with dissipation (QCM-D) and total internal reflection fluorescence (TIRF) microscopy. In doing so, the mechanism of MSN adsorption onto biologically relevant lipid bilayers was quantified, which allowed for direct comparisons to be made with cellular uptake and antibacterial efficacy studies. Such biophysical insights derived from this study can be harnessed for the optimisation of porous biomaterials in delivering bioactive molecules to epithelial cells.

## 2. Materials and Methods

All materials were sourced from Sigma-Aldrich (Castle Hill, NSW Australia; Stockholm, Sweden) unless otherwise specified.

### 2.1. Fabrication of Mesoporous Silica Nanoparticles (MSN)

#### 2.1.1. Synthesis of MSN

Mesoporous silica nanoparticles (MSN) were prepared via the synthesis method developed by Subramaniam et al. [14], using cetyltrimethylammonium bromide (CTAB) as a templating agent, tetraethyl orthosilicate (TEOS) as a silica source, hexane as the hydrophobic component and L-lysine as a catalyst. CTAB (800 mg) was mixed with L-lysine (180 mg) prior to forming an emulsion with Milli-Q (248 g) and hexane (60 g for 40 nm particles; 120 g for 80 nm particles), via vigorous stirring for 1 h at 70 °C. TEOS (8 g) was added to the emulsion, which continued stirring for 20 h at the same temperature. The suspension was cooled at room temperature and oven dried for 3 h at 80 °C. In this study, the surfactant was removed either via (i) calcination, by increasing the temperature from room temperature to 650 °C for 8 h and maintaining this temperature for a further 6 h, to create ‘calcined MSN’ (MSNc); or, (ii) solvent extraction, where the dried powder was resuspended in a mixture of 90 mL of methanol and 10 mL of 12 M HCl and left to reflux at 70 °C overnight, to create ‘extracted MSN’ (MSNe). The suspension was centrifuged at 29,060 *g* for 10 min. After washing with methanol, the particles were refluxed again to ensure all of the organic material was removed. After centrifugation and washing, the particles were oven dried.

#### 2.1.2. Rhodamine B Loading into MSN

MSN were dispersed in Milli-Q at a concentration of 1 mg/mL via sonication. Rhodamine B stock (1 mg/mL in Milli-Q) was added to the dispersed particles at a ratio of 1:100 and left to stir for 2 h. The particles were collected by centrifugation at 29,060 rcf for 10 min and washed three times with Milli-Q to remove any rhodamine B freely dispersed within the aqueous media (i.e., not encapsulated within MSN).

#### 2.1.3. Rifampicin Loading into MSN

MSN (2 mg) were added to 4 mg rifampicin solution in methanol (2 mL). The suspension was stirred at 500 rpm for 24 h, subsequently centrifuged, washed and oven dried overnight. Non-encapsulated rifampicin within the supernatant was recovered and quantified via UV–Vis spectroscopy at a wavelength of 254 nm to determine the loading capacity and encapsulation efficiency, as previously described by Subramaniam et al. [14].

### 2.2. Physicochemical Characterization of MSN

#### 2.2.1. Particle Sizing and Structure

MSN were suspended in ethanol at a concentration of 1 mg/mL via sonication for 1 h. Samples (5 µL) were transferred to copper grids and left to air dry for > 30 min to allow for complete ethanol evaporation. Transmission electron microscopy (TEM) analysis was performed using a JEM-1200 EX II (JEOL) at 120 kV accelerating voltage.

#### 2.2.2. Nitrogen Adsorption/Desorption Isotherms

Nitrogen isotherms were measured at liquid nitrogen temperature using a Micromeritics TriStar II volumetric adsorption analyzer (Micromeritics Instrument Corporation, GA, USA). Prior to measuring, the MSN were outgassed for 3 h at 200 °C. The Brunauer–Emmett–Teller (BET) equation was used to calculate the surface area from the adsorption data obtained in the relative pressure range of 0.05 to 0.3. The total pore volume was calculated from the amount of gas adsorbed at 0.91 (*P*/*P_0_*) and the pore size distribution curves were derived using the Barrett–Joyner–Halenda (BJH) method.

#### 2.2.3. Thermogravimetric Analysis (TGA)

The relative degree of surfactant remaining within untreated MSN (MSNut, i.e., no surfactant removal), MSNc and MSNe was determined by heating the MSN at a scanning rate of 10 °C/min from 20 to 600 °C, under nitrogen purging. CTAB completely decomposed by 500 °C, whilst the silica remained thermally stable under these conditions. The amount of CTAB remaining within the MSN was determined by the weight loss accounting for trace amounts corresponding to residual water moisture.

#### 2.2.4. Fourier Transform Infra-Red Attenuated Total Reflection (FTIR-ATR) Spectroscopy

Infrared spectra of CTAB, MSNut, MSNc and MSNe were recorded using a Perkin Elmer, Spectrum Two spectrometer (Waltham, MA, USA) using a universal ATR over 600–4000 cm^−1^.

### 2.3. Quartz Crystal Microbalance with Dissipation (QCM-D) Studies

QCM-D measurements were performed on silicon dioxide-coated QSX 303 QCM-D sensors mounted in a Q-Sense E4 system (Biolin Scientific AB, Gothenburg, Sweden). The sensor and solution chamber were maintained at 37 ± 0.1 °C for the duration of the experiments and the third harmonic was recorded. The sensors were first flushed with TRIS buffer (125 mM NaCl (Sigma Aldrich, Stockholm, Sweden), 10 mM TRIS (Merck, Stockholm, Sweden), 1 mM Na_2_EDTA (Sigma Aldrich), adjusted to pH = 7.4 using HCl) at a flow rate of 50 µL/min. Supported lipid bilayer (SLB) formation was monitored for ~10 min by incubating 1-palmitoyl-2-oleoyl-sn-glycero-3-phosphocholine (POPC; Avanti Lipids Inc., Alabaster, AL, USA) vesicles (0.1 mg/mL in TRIS buffer) at continuous flow. Following rinsing, the SLB was exposed to various MSN (concentration = 10^5^ particles/mL in TRIS buffer), while concurrently monitoring changes in frequency (Δf) and dissipation (ΔD).

### 2.4. Total Internal Reflection Fluorescence (TIRF) Microscopy Studies

#### 2.4.1. Preparation of Semi-Native Lipid Vesicles

Synthetic vesicles were prepared using a lipid film hydration and extrusion method, where POPC was dissolved in chloroform, which was then evaporated at the bottom of a round bottom flask under vacuum for > 2 h to remove any trace of the solvent. POPC was rehydrated with TRIS buffer for at least 3 h to obtain a lipid concentration of 1 mg/mL. The vesicle solution was extruded through a 50 nm polycarobante membrane (Whatman, Maidstone, UK) 11 times using a mini extruder (Avanti Lipids Inc., Alabaster, AL, USA). Extruded vesicles were stored at 4 °C until use.

Native membrane vesicles (NMVs) were prepared using a SF9 cell line via a detergent-free preparation protocol, as previously described by Pace et al. [29,30]. NMVs were mixed with POPC vesicles at a ratio of 1:5 to form semi-native membrane vesicles (sNMV).

#### 2.4.2. Preparation of Fluorescent Tracer Vesicles

Tracer vesicles were prepared by mixing 99 mol% POPC with 1 mol% 1,2-dimyristoyl-sn-glycero-3-phosphoethanolamine-N-(7-nitro-2-1,3-benzoxadiazol-4-yl) (NBD-PE; Avanti Lipids Inc., Alabaster, AL, USA) and following the lipid film hydration and extrusion method.

#### 2.4.3. Formation of a Semi-Native Supported Lipid Bilayer (snSLB)

Total internal reflection fluorescence (TIRF) microscopy was conducted on an inverted Eclipse Ti-E microscope (Nikon Corporation, Minato City, Japan) that was equipped with a Perfect Focus System (PFS), a CFI Apo TIRF 100x oil objective (NA 1.49), a high-pressure mercury lamp and an Andor Neo SCC-01322 sCMOS camera (Andor Technology, Belfast, UK). A snSLB was formed on a flat glass substrate (0.13–0.16 mm thickness). sNMV vesicles were incubated with NBD-PE tracer vesicles at a ratio of 1/100, with 10 µL added to custom made PDMS wells with a volume of ~ 50 µL. A FITC filter set (Semrock, Sandwich, IL, USA) was used for visualising the snSLB or POPC/NBD-PE vesicles. snSLB formation was confirmed by observing fluorescent recovery after photobleaching (FRAP), i.e., by bleaching the NBD-PE tracer lipids with a Kr-Ar mixed gas ion laser (Stabilite 2018, Spectra-Physics Lasers, Mountain View, CA, USA) at a wavelength of 531 nm. The diffusivity of NBD-PE within the lipid membrane was determined using a custom written analysis software in MATLAB R2017B (MathWorks, 2017, Natick, MA, USA), as described by Jonsson et al. [31] The snSLB was then subjected to rinsing with TRIS buffer until the majority of unbound vesicles were removed.

#### 2.4.4. Monitoring of MSN Adsorption onto snSLB

A volume of 10 µL of rhodamine-labelled MSN dispersion (concentration = 10^5^ particles/mL) was added to the rinsed snSLB, and adsorption was monitored with TIRF microscopy, imaging at 10 frames per second for 2 min, using a rhodamine filter set (TRITC, Semrock, Sandwich, IL, USA). The number of particles adsorbed onto the snSLB was quantified using a custom written analysis software in MATLAB R2017B (MathWorks, 2017, Natick, MA, USA) and the fluorescent intensity of MSN aggregates was analysed using ImageJ (Fiji, 2017, Lexington, KY, USA) [32].

### 2.5. In Vitro Cellular Uptake Studies

#### 2.5.1. Cellular Uptake of MSN Particles with Flow Cytometry

The cellular uptake of rhodamine B-labelled MSN was investigated using fluorescence-activated cell sorting (FACS), in which the Caco-2 cells were seeded at 5 × 10^4^ cells/mL in Dulbecco’s Modified Eagle’s Medium (DMEM) medium. The cells were incubated for 24 h at 37 °C with 5% CO_2_ for cell attachment. Following incubation, the medium was removed, and the cells were treated with pure rhodamine B and various rhodamine B-MSN (rhodamine B concentration of 50 µg/mL) in DMEM. Following incubation for 4 h, the supernatant was discarded, and the cells were gently washed 3× with ice-cold PBS to remove any extracellular particles. Following rinsing, the cells were centrifuged at 600 *g* for 5 min to obtain the cell pellet. The supernatant was discarded, and the pellet was resuspended in 2% paraformaldehyde (PFA) and incubated for 15 min at room temperature, prior to centrifugation and washing in triplicate. The rinsed cells were subsequently transferred into FACS tubes. The fluorescence was analysed using Accuri C6 Plus flow-cytometer (BD Biosciences, Franklin Lakes, NJ, USA).

#### 2.5.2. Cellular Uptake of MSN Particles with Confocal Microscopy

Confocal fluorescence imaging was performed with a Zeiss Elyra PS-1 Super Resolution Microscope (Oberkochen, Germany), using a white-light laser source and a 60× objective. Briefly, Caco-2 cells (seeding density of 1 × 10^5^ cells/well) were incubated with rhodamine B-MSN at a concentration of 50 µg/mL in DMEM. After 4 h incubation, the supernatant was discarded, and the cells were washed 3x with PBS and fixed with 4% paraformaldehyde. Before imaging, the nuclei were stained with 4′,6-diamidino-2-phenylindole (DAPI) and imaged at an emission wavelength of 461 nm (excitation wavelength 358 nm) and the cell membrane was stained with Alexa-488 at an emission wavelength of 525 (excitation wavelength of 490 nm) which appeared as blue and green, respectively.

### 2.6. In Vitro Cell Viability Studies

An intracellular infection assay was performed in Caco-2 cells by modifying a method developed by Clemens et al. [33]. Caco-2 cells were seeded at 1 × 10^5^ cells/mL and incubated for 24 h (37 °C, 5% CO_2_). SCV *S. aureus* overnight cultures were prepared and diluted to a multiplicity of infection (MOI) of 10:1. After centrifugation of the bacterial suspension (600 *g*, 10 min, room temperature), the supernatant was carefully discarded and the pellet was redispersed in DMEM media. DMEM inoculum (1 mL) was added to the cells and incubated for 1 h. Following incubation, the cells were rinsed 3× to remove any extracellular bacteria. Confocal imaging was used to confirm the presence of *SCV S. aureus* within the Caco-2 cells, by staining both the bacteria and cell nucleus with DAPI and the cell membrane with Alexa-488.

Infected Caco-2 cells were incubated with 1 mL of fresh serum-free DMEM containing either rifampicin or rifampicin-loaded MSN at a rifampicin concentration of 0.5 µg/mL. Following a 4 h incubation, the medium was removed, and the cells lysed were by 0.1% Triton-X in PBS. The cells were then transferred to Eppendorf tubes, serially diluted, and plated on Tryptic Soy Agar (TSA) for 15 h at 37 °C. To confirm the number of bacteria in the inoculum, serial dilutions of the DMEM inoculum were also plated on TSA.

### 2.7. Statistical Analysis

The experimental data were analysed statistically using a Student’s *t*-test (unpaired). Values are reported as the mean ± standard deviation, and the data were considered statistically significant when *p* < 0.05.

## 3. Results and Discussion

### 3.1. Synthesis and Characterization of MSN

**Figure 1:**
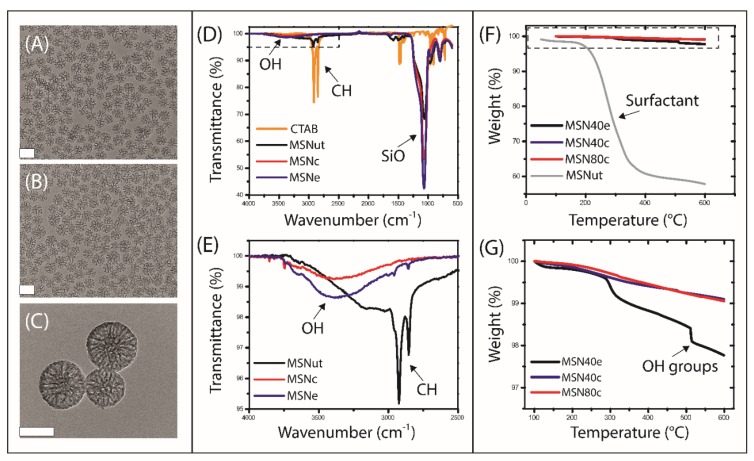
Transmission electron micrographs of (**A**) MSN40c, (**B**) MSN40e and (**C**) MSN80c, where scale bars represent 50 nm. (**D**) Infra-Red Attenuated Total Reflection (IR-ATR) spectra of templating surfactant (CTAB; yellow curve), MSNut (black curve), MSNc (red curve) and MSNe (blue curve), with (**E**) inset of localized region highlighting the presence of OH and CH groups in various MSN. (**F**) Thermogravimetric curves for MSNut (grey curve), MSN40e (black curve), MSN40c (blue curve) and MSN80c (red curve), with (**G**) inset highlighting the degradation within treated MSN. MSN, mesoporous silica nanoparticles.

Hiroshima mesoporous material (HMM)-type mesoporous silica nanoparticles (MSN) were synthesised with varying particle sizes and hydrophilicities by varying the organic solvent concentration and surfactant extraction process (i.e., calcination or solvent extraction), respectively (Figure 1). Transmission electron micrographs revealed that spherical mesoporous particles were fabricated with mean particle sizes of 47.0 ± 7.0 nm (MSN40c and MSN40e) and 84.1 ± 17.4 nm (MSN80c) (based on imaging analysis with Fiji ImageJ [32]) and with mean pore diameters of 11.7 and 7.46 nm (based on BJH pore size distribution analysis; Supplementary Materials Figure S1), respectively (Table 1). ATR-FTIR was performed to validate the removal of the cytotoxic templating surfactant, CTAB [34,35], from the MSN, which also highlighted key differences in the surface chemistry of MSN treated with solvent extraction and calcination (Figure 1D,E). Evidence of CTAB removal from both calcined and solvent-extracted MSN was displayed through the absence of the C–H stretching of methylene groups at ~2800 cm^−1^. In contrast, untreated MSN (i.e., no surfactant removal; MSNut) clearly displayed the C–H stretching associated with the methylene groups in CTAB. However, TGA demonstrated that CTAB was not completely removed from the treated MSN, with a minor fraction (< 1 wt%) of CTAB remaining after calcination and extraction (Figure 1G).

While no significant differences were observed with respect to the zeta potential of MSN (Table 1), differences in IR spectra highlighted variations in surface chemistry between calcined and solvent-extracted MSN, specifically with regards to the increased level of hydroxyl/silanol group stretching at ~3500 cm^−1^ for the solvent-extracted MSN (MSN40e). TGA further confirmed the presence of additional -OH groups in MSN40e, compared to MSN40c and MSN80c, where -OH groups were shown to decompose at ~500 °C (Figure 1F,G). This is in agreement with previous studies that have shown that calcination of mesoporous silica results in the condensation of Si–OH bonds, resulting in a higher ratio of Si–O–Si bonds [36,37,38]. In contrast, solvent extraction has been shown to be a suitable method of surfactant removal that maintains a high silanol group density on the mesoporous silica surface, thus maintaining the hydrophilicity and potential biocompatibility of the silica particles [39,40,41,42]. Rifampicin loading capacities were shown to be dependent on MSN size and surface chemistry, with drug loading increasing as a function of increasing particle size and hydrophobicity (Table 1). BJH pore size distribution analysis revealed that the pore volume of MSN40 was 1.6-fold greater than MSN80, with both particle sets having equivalent specific surface areas (Supplementary Materials Figure S1). Since drug loading was greatest in MSN80c, it can be assumed that loading was more dependent on particle size and hydrophobicity, rather than pore volume.

### 3.2. Cell Viability and Caco-2 Uptake of MSN

**Figure 2:**
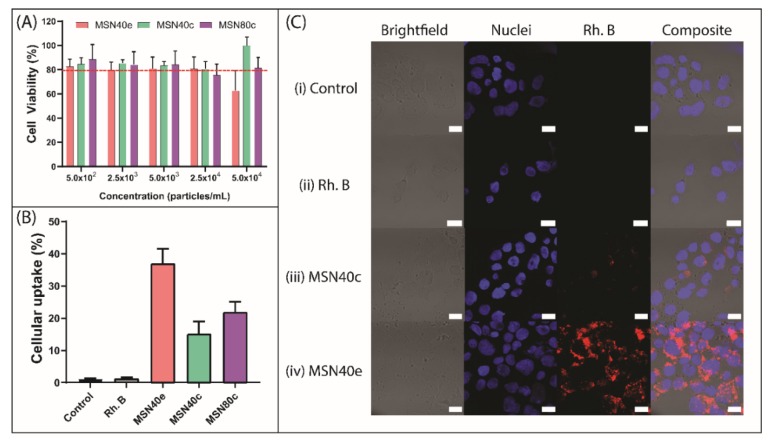
(**A**) Cell viability of Caco-2 cells after 24 h incubation with MSN40e (red bars), MSN40c (green bars) and MSN80c (purple bars). (**B**) Caco-2 uptake of rhodamine B (grey bar), Rh-MSN40e (red bar), Rh-MSN40c (green bar) and Rh-MSN80c (purple bar) after incubation for 4 h, as measured by fluorescence-activated cell sorting (FACS) (mean ± SD, n = 3; *p* < 0.05 for MSN40e compared to both MSN40c and MSN100c). (**C**) Laser scanning confocal micrographs for Caco-2 cells treated with (i) no treatment, (ii) rhodamine B, (iii) Rh-MSN40c and (iv) Rh-MSN40e. Nuclei were stained with DAPI (blue) and MSN were stained with rhodamine B (red). Scale bars = 20 µm.

Cytotoxicity screening of MSN was performed using the MTT assay in Caco-2 cells, where MSN exhibited cell viability ≥ 80% at concentrations of up to 2.5 × 10^4^ particles/mL (Figure 2A). Cellular survival of 80% is considered the accepted threshold for cell viability [43] and, thus, MSN at concentrations ≤ 2.5 × 10^4^ particles/mL can be considered non-toxic. At 5.0 × 10^4^ particles/mL, MSN40e triggered a cellular viability of 62.6 ± 16.5% and can therefore be considered marginally toxic at this concentration. This could be attributed to either (i) a higher density of silanol groups present on MSN40e, compared to MSN40c and MSN80c, since silanol groups have been shown to interact with cellular membrane components, triggering cell lysis and death [44,45,46,47], or (ii) increased particle uptake for MSN40e (Figure 2B) and, thus, increased exposure to CTAB residues within the MSN. However, since all particle concentrations used in the cellular uptake and intracellular infection assays were below 2.5 × 10^4^ particles/mL, the MSN can be safely considered non-toxic to the Caco-2 cell line.

The intracellular uptake of MSN particles into Caco-2 cells was observed by encapsulating the poorly permeable dye, rhodamine B, into the porous cavities, for quantification using flow cytometry (Figure 2B) and qualitative assessment using confocal fluorescence microscopy (Figure 2C) [48]. For all MSN, the fluorescence intensity associated with cellular uptake was significantly greater than for the rhodamine B solution, which only achieved 1.2 ± 0.4% uptake into Caco-2 cells, thus indicating that rhodamine B is incapable of permeating the cell membrane through diffusion or endocytosis. In contrast, MSN particles achieved > 15% cell internalization, demonstrating their ability to be endocytosed by the Caco-2 cells. No significant difference was observed for the uptake between the two groups of calcined MSN, with MSN40c and MSN80c exhibiting 15.1 ± 3.9% and 21.9 ± 3.2% uptake, respectively. This highlights that varying the MSN size from ~40 to ~80 nm does not significantly alter uptake into Caco-2 cells. Previous in vitro studies have highlighted that maximum cellular uptake is achieved within the 10–100 nm size range, since nanoparticles are capable of recruiting and binding to a sufficient number of membrane receptors at this particle diameter to drive the membrane-wrapping and pinocytic process [49,50,51,52].

MSN40e exhibited enhanced Caco-2 uptake in contrast to both the dye solution and the calcined MSN, with 36.9 ± 4.6% of particles being internalized after 4 h incubation (Figure 2B). The contrasting ability for MSN prepared via solvent extraction, versus calcination, was further highlighted using confocal microscopy, where dense areas of rhodamine B (red regions) surrounded the nucleus (blue regions) of the Caco-2 cells (Figure 2C). Comparatively, only a minor fraction of dye can be observed within the cells treated with MSN40c. This is in accordance with previous studies that have highlighted that an increase in silanol group density drives the interaction between silica nanoparticles and the cell membrane, thus triggering an increase in cellular uptake [46,53]. However, a fine balance exists between increasing cellular uptake and limiting cytotoxicity of MSN, since increasing silanol concentrations also triggers a reduction in cell viability (Figure 2A) [46].

### 3.3. Biophysical Analysis of MSN Adsorption onto Biologically Relevant Lipid Bilayers

**Figure 3:**
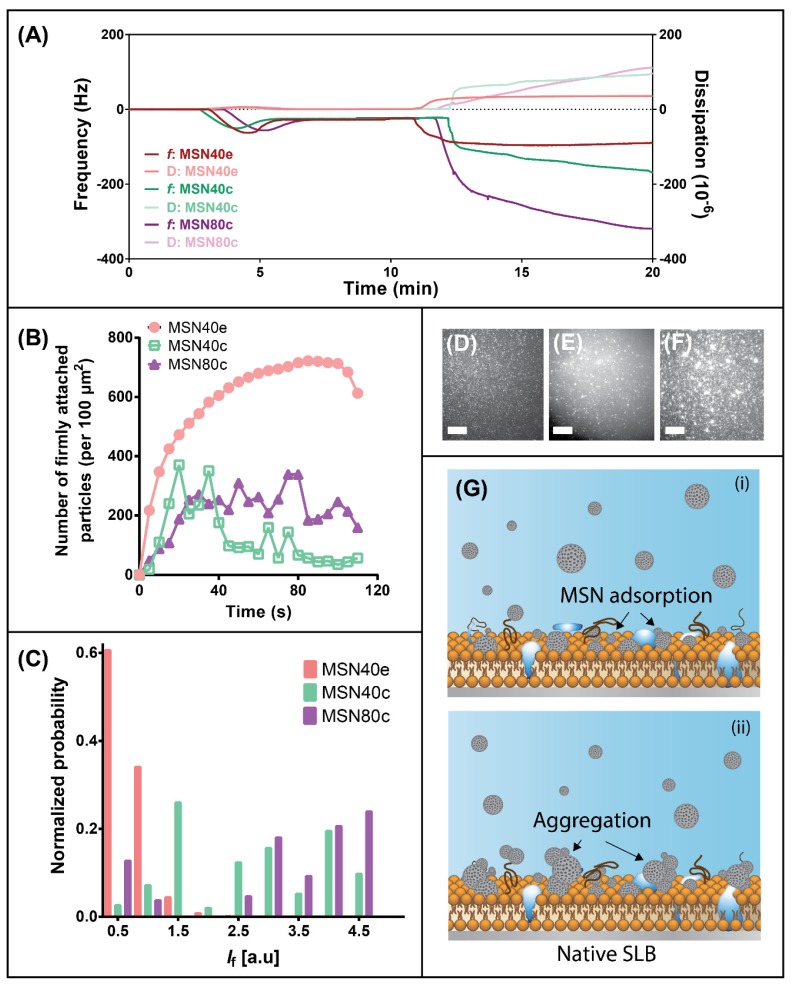
(**A**) Frequency (dark curves; right axis) and dissipation (light curves; left axis) profiles for adsorption of MSN40e (red curves), MSN40c (green curves) and MSN80c (purple curves) onto a supported lipid bilayer (SLB), using quartz crystal microbalance with dissipation (QCM-D). (**B**) The time-dependent change in the number of counted MSN particles firmly adsorbed onto a semi-native SLB for MSN40e (red circles), MSN40c (green empty squares) and MSN80c (purple triangles). (**C**) The normalized mean fluorescence (*I*_f_) of MSN particles/aggregates adsorbed onto a semi-native SLB after 2 min for MSN40e (red bars), MSN40c (green bars) and MSN80c (purple bars). Representative total internal reflection fluorescence (TIRF) micrographs for (**D**) MSN40e, (**E**) MSN40c and (**F**) MSN80c after 2 min adsorption onto a semi-native SLB. Scale bars = 10 µm. (**G**) Schematic representation of the (i) adsorption and (ii) aggregation mechanism of MSNc particles on a semi-native SLB.

Quartz crystal microbalance with dissipation (QCM-D) was used to qualitatively assess the impact of MSN surface chemistry and size on their interactions with biologically relevant supported lipid bilayers in order to identify possible differences in the cellular uptake behavior of each MSN. By assessing changes in frequency, *f*, and dissipation, D, it is possible to deduce relative changes in the adsorption of species on a supported lipid bilayer (SLB) [54]. Evidence of SLB formation on the planar silica surface was indicated at ~ 4–5 min for each sample, whereby a decrease in *f* and increase in D due to POPC vesicle adsorption was followed by an increase in *f* and decrease in D caused by vesicle rupturing and bilayer formation (Figure 3A). The final Δ*f* and ΔD of ~−27 Hz and 0.1 × 10^−6^ (third overtone), respectively, is consistent with previous findings [29,30]. After rinsing, the SLB was exposed to MSN dispersions, where adsorption of MSN was evidenced by rapid decreases in *f* and increases in D due to the viscoelastic nature of nanoparticle adsorption (at *t* ≈ 10 min). After 3 min adsorption, the change in frequency and dissipation plateaued for MSN40e, indicating that the SLB was likely saturated with silica nanoparticles. In contrast, for calcined particles, *f* and D continue to decrease and increase, respectively, in a time-dependent manner until the completion of the experiment, indicating that a highly viscoelastic layer of MSN adsorbs onto the SLB surface. Changes in frequency and dissipation were greatest for MSN80c, which can be attributed to adsorption of particles twice the size of MSN40.

Total internal reflection fluorescence microscopy was used to further elucidate the different MSN adsorption mechanisms on lipid bilayers. To achieve this, a semi-native supported lipid bilayer (snSLB) composed of 20% w/w native membrane vesicles was adsorbed onto a planar silica surface, since this presents a more accurate mimic for the cellular membrane due to the presence of native lipids, proteins and carbohydrates. TIRF microscopy operates by illuminating a sample at an angle of incidence that triggers light to internally reflect at the interface between a material with varying refractive indices (known as the critical angle) [55]. The evanescent wave that forms as a result of total internal reflection decays exponentially into the material with a lower refractive index (in this case, the sample) in a distance-dependent manner [56]. Ultimately, this allows the depth of penetration of the evanescent wave to be limited to ~100–200 nm from the glass substrate surface, allowing for the events at the substrate-supported lipid membrane to be monitored [54]. For this application, TIRF microscopy was harnessed to discriminate and quantify only the MSN that adsorbed to the snSLB, allowing for the derivation of new understanding with regards to the impact of particle size and surface chemistry on membrane adsorption.

The formation of a snSLB was first achieved by depositing semi-native membrane vesicles, labelled with a fraction of tracer vesicles containing NBD-PE (1 mol%), onto a smooth, hydrophilic silica surface, as previously demonstrated by Pace et al. [29,30]. Semi-native membrane vesicles were used in this study, since the presence of membrane proteins and carbohydrates and native lipids serves as a better mimic for real cell membranes [29,30]. The presence of a small fraction of dye-labelled lipids allowed for real-time monitoring of vesicle adsorption and rupturing, and the subsequent bilayer formation. Fluorescent recovery after photobleaching (FRAP) was performed to validate complete formation of a mobile and intact snSLB (Supplementary Materials Figure S2) [31]. After washing excess membrane vesicles from the bulk media, the snSLB was incubated with rhodamine-loaded MSN and adsorption was monitored via TIRF (Figure 3D–F).

Incubation of the snSLB with MSN40e revealed a time-dependent increase in the number of particles that firmly attached to the lipid bilayer surface, until a maximum of ~610 particles/100 µm^2^ was reached (Figure 3B). After this point, a decrease in the number of counted particles attached to the surface was realised. It must be noted that the analysis approach employed cannot discriminate between individual nanoparticles once aggregated on the membrane surface. That is, an aggregate of many MSN is counted as one particle. Subsequently, it can be assumed that the decrease in the number of counted particles after 100 s was not due to particle desorption, but rather particle aggregation of the individual MSN40e/small clusters on the snSLB surface. Thus, it is hypothesised that the snSLB surface became saturated with individual MSN40e/small aggregates, which then continued to aggregate as additional MSN40e diffuse towards the surface, triggering a reduction in the analysed number of particles attached to the surface.

The aggregation of MSN was increasingly evident for MSN40c and MSN80c, which both exerted complex particle adsorption profiles, whereby increases in particle adsorption were followed with rapid decreases in particle counts (Figure 3B). Since the maximum number of counted particles/aggregates adsorbed onto the snSLB surface was reduced for the calcined MSN samples, it does not indicate that fewer individual MSN particles diffused to the surface, but rather the more hydrophobic MSN had an increased propensity for particle aggregation. These findings correlate well with QCM-D analysis, whereby the continual decrease in *f* and increase in D can be attributed to the formation of large aggregates on the SLB surface. Interestingly, evidence of MSN40e aggregation on the SLB is not apparent for MSN40e during QCM-D experiments, which suggests that aggregation may be influenced by the flow of media (QCM-D was performed under flow conditions, whereas TIRF was static) or the presence of proteins and carbohydrates within the SLB. To further indicate aggregation on the snSLB surface, analysis of adsorbed MSN fluorescence intensity (*I*_f_) highlighted an increase in mean intensity for both calcined MSN, compared to MSN40e (Figure 3C). Since fluorescence intensity has been shown to scale with particle size [57], it can be concluded that MSN40c and MSN80c formed significantly larger aggregates on the snSLB surface, compared to MSN40e, which is hypothesised to be driven by hydrophobic forces between individual nanoparticles (Figure 3G).

The increased aggregability of MSN40c and MSN80c correlates well with a reduction in Caco-2 cell uptake, since the particle size presented to the cell membrane was expected to be greater than the individual particles, thus exceeding the size required for pinocytosis (< 500 nm) [58] and limiting the interaction between the MSN surface and cell membrane receptors that are responsible for driving endocytic uptake [59,60]. Previous studies have highlighted that MSN uptake into epithelial cells, such as Caco-2 cells, is highly limited by their ability to remain monodispersed and avoid aggregation in biological fluid [22]. While these studies have not been performed in biological fluid and are therefore not ideal replicates for simulating particle uptake in vivo, the biophysical interactions observed in this study suggest that the propensity for nanoparticle aggregation between calcined MSN may retard their uptake into epithelial cells, through endocytic pathways, to a greater degree than MSN prepared through solvent extraction. It is important to note that further studies characterizing endocytosis pathways, through receptor knockdown approaches, are required to fully elucidate the differences in uptake between the various MSN.

### 3.4. Antibacterial Efficacy of Rifampicin-Loaded MSN against Intracellular Pathogens

**Figure 4:**
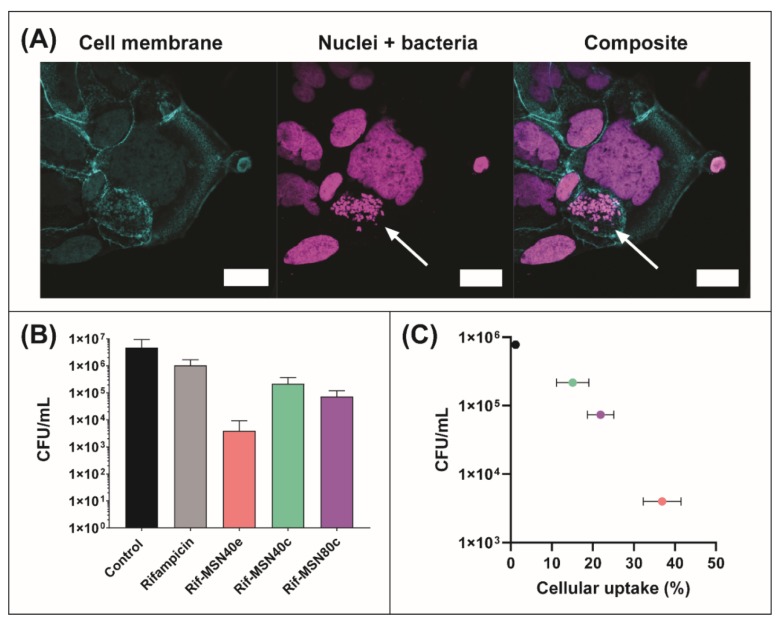
(**A**) Laser scanning confocal fluorescence micrographs of SCV *S. aureus*-infected Caco-2 cells. SCV *S. aureus* and nuclei were labelled with DAPI (magenta) and the cell membrane was labelled with Alexa-488 (cyan). Intracellular pathogens are highlighted by the arrow. (**B**) Efficacy of the following rifampicin formulations in the reduction of intracellular SCV *S. aureus* within Caco-2 cells: rifampicin solution (grey bar), MSN40e (pink bar), MSN40c (green bar), and MSN80c (purple bar), relative to a control group (i.e., no treatment; black bar). (**C**) Colony forming units of intracellular pathogens as a function of cellular uptake of each formulation (from data already presented).

Caco-2 cells were infected with small colony variants of *S. aureus* (SCV *S. aureus*) to serve as an intracellular infection model for this study, since increasing evidence is indicative of these subpopulations existing as a problematic source of infection due to their ability to survive within the intracellular environment of mammalian cells, including within the GI tract [61,62,63]. SCV *S. aureus* replicate slowly, creating subpopulations that are 10-fold smaller and phenotypically different compared to the parent strain, which is hypothesised to be a driving force for their intracellular internalization and clinically challenging nature [64]. Unlike their parent strains, SCV acquire two distinct metabolic characteristics, which leads to phenotypic differences, such as defects in electron transport and reduced energy (ATP) usage, triggering the slower growth and smaller colony formation [64,65]. Here, the intracellular internalization of SCV *S. aureus* was confirmed and visualised using confocal fluorescence imaging, as shown in Figure 4A.

The rifampicin concentration used throughout the intracellular efficacy assay was 0.5 µg/mL, which was equivalent to 4-fold greater than the Minimum Inhibitory Concentration (MIC) of SCV *S. aureus* (determined previously to be 0.125 µg/mL) [16]. Infected Caco-2 cells treated with a rifampicin solution showed only a minor reduction in the number of colony forming units (CFU) of SCV *S. aureus*, after 4 h incubation, which was determined to be statistically insignificant to the control group (i.e., no treatment) (Figure 4B). In doing so, this further validated the inability for rifampicin to permeate the cell membrane and be internalized within Caco-2 cells, therefore, exerting limited efficacy in the reduction of intracellular pathogens, as demonstrated previously for infected macrophages [14,16]. In contrast, treatment of infected Caco-2 cells with all rifampicin-loaded MSN resulted in a statistically significant decrease in SCV *S. aureus* CFU, when compared to the control group (Figure 4B). Rifampicin-loaded MSN40e revealed the greatest antibacterial activity, with a > 2-log reduction in CFU (i.e., ~5 × 10^6^ CFU/mL reduced to ~5 × 10^4^ CFU/mL), which equates to an extermination of > 99.9% of intracellular pathogens. It must be noted that previous studies have highlighted that rifampicin release from MSN prior to cellular internalization is minimal [14], and therefore, the enhanced antibacterial efficacy is attributed to enhanced localized concentrations of rifampicin at the site of infection. Furthermore, while pore volume and diffusional path length have been shown to impact on the transport of bioactive molecules to and from porous silica particles [66], the differences in these two parameters are minimal in this study, and are therefore not considered to be major factors in controlling rifampicin release.

Importantly, antibacterial efficacy log-linearly correlated with cellular uptake of formulations in Caco-2 cells. Thus, this highlights that the improved internalization of MSN translated to an increased antibiotic concentration localized at the site of pathogen confinement; thus, increasing the susceptibility of bacteria to rifampicin. This is in agreement with previous studies that have shown that increasing antibiotic concentrations within macrophages infected with SCV *S. aureus* also leads to an increase in the extent of pathogen eradication [14,16]. Furthermore, this suggests that surface chemistry is a more important factor for controlling drug delivery into Caco-2 cells.

Ultimately, this study provides further preclinical proof of concept and reveals new opportunities for repurposing conventional antibiotics, through their encapsulation within nanocarrier systems that are known to be efficiently internalized within infected cells, in order to increase their antibacterial activity against pathogens that shield themselves within the intracellular environment. Moreover, it is stipulated that more advanced surface modifications should be applied to MSN to optimise cellular uptake and antibacterial efficacy, while limiting cytotoxicity. By attributing focus to optimising this antibiotic delivery approach in key infection models and by validating this formulation approach within in vivo intracellular infection models, it is expected that repurposing antibiotics in such a manner will reduce the clinical dose required to effectively kill intracellular pathogens.

## 4. Conclusions

Mesoporous silica nanoparticles with varying surface chemistries and particle sizes were shown to be effective vehicles for delivering the antibacterial drug, rifampicin, to epithelial cells infected with *S. aureus*. For the particle sizes tested (~40 and ~80 nm), surface chemistry was shown to be the most significant regulator of MSN uptake, with more hydrophilic particles (prepared using solvent extraction) demonstrating a > 2-fold increase in Caco-2 cell uptake, compared to calcined MSN. QCM-D and TIRF microscopy revealed that calcined MSN had an increased tendency to aggregate on biologically relevant lipid membranes, which was expected to delay particle uptake through endocytic pathways. The efficacy of rifampicin-loaded MSN correlated well with cellular uptake studies, with hydrophilic MSN exerting the greatest antibacterial activity, reducing SCV *S. aureus* colony forming units in Caco-2 cells by ~ 2.5-fold. Thus, this study highlights the ability to regulate the antibacterial efficacy of rifampicin-loaded MSN through simple synthesis modifications, which ultimately alter the uptake mechanisms within epithelial cells. However, to assess the efficacy of this treatment approach further, future focus should be attributed to developing intracellular infection animal models and comparing/correlating in vitro performance with in vivo findings in an attempt to translate antibacterial formulations to the clinic.

## Figures and Tables

**Table 1:** Physicochemical properties of MSN.

Particle	Surfactant Extraction Protocol	Mean Particle Size (nm)	Mean Pore Width (nm)	Specific Pore Volume (cm^3^/g)	Specific Surface Area (m^2^/g)	Zeta Potential (mV)	Drug Loading (% w/w)
MSN40e	Solvent extraction	47.0 ± 7.0	11.7	1.30	483	−15.1 ± 5.4	28.9
MSN40c	Calcination	47.0 ± 7.0	11.7	1.30	483	−13.6 ± 6.3	33.6
MSN80c	Calcination	84.1 ± 17.4	7.46	0.81	460	−14.9 ± 4.7	38.2

## Supplementary Materials

The following are available online at https://www.mdpi.com/2079-4991/10/4/815/s1, Figure S1: Nitrogen adsorption/desorption isotherms and BJH pore size distribution of MSN. Figure S2: Total internal reflection micrographs of semi-native SLB.
