# Supplementary material for: Enhancing the Cellular Uptake and Antibacterial Activity of Rifampicin through Encapsulation in Mesoporous Silica Nanoparticles
Source: Nanomaterials (Basel). 2020 Apr 24;10(4):815. doi: 10.3390/nano10040815 (PMC7221943; doi:10.3390/nano10040815)
Supplement: Supplementary file 1 [file nanomaterials-10-00815-s001.pdf]

nanomaterials

Supplementary Information

Enhancing the Cellular Uptake and Anti-Bacterial Activity of Rifampicin through Encapsulation in Mesoporous Silica Nanoparticles

Paul Joyce [1], Hanna Ulmefors [2,3], Sajedeh Maghrebi [2,3], Santhni Subramaniam [2,3], Anthony Wignall [2,3], Silver Jõemetsa [1], Fredrik Höök [1] and Clive A. Prestidge [2,3,*]

[1] Department of Physics, Chalmers University of Technology, SE-412 96 Gothenburg, Sweden; paul.joyce@unisa.edu.au (P.J.); silver@chalmers.se (S.J.); fredrik.hook@chalmers.se (F.H.)
[2] School of Pharmacy & Medical Sciences, University of South Australia, Adelaide, South Australia 5090, Australia; hanna.gustafsson@chalmers.se (H.U.); sajedehsadat.maghrebi@mymail.unisa.edu.au (S.M.); santhni.subramaniam@mymail.unisa.edu.au (S.S.); anthony.wignall@unisa.edu.au (A.W.)
[3] ARC Centre of Excellence in Bio-Nano Science and Technology, University of South Australia, Adelaide, South Australia 5090, Australia
* Correspondence: clive.prestidge@unisa.edu.au

Received: 26 March 2020; Accepted: 20 April 2020; Published: date

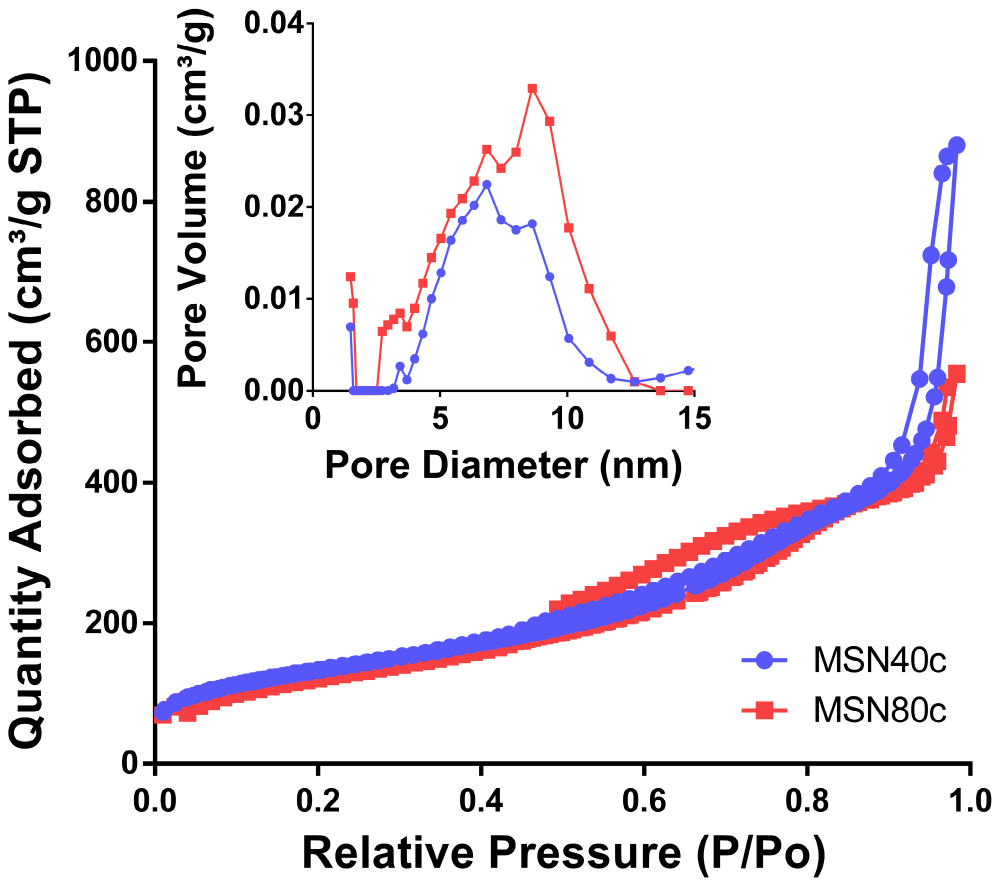

Figure S1. Nitrogen adsorption/desorption isotherms for MSN40c (blue dots) and MSN80c (red squares), with corresponding BJH pore size distribution of MSN (inset).

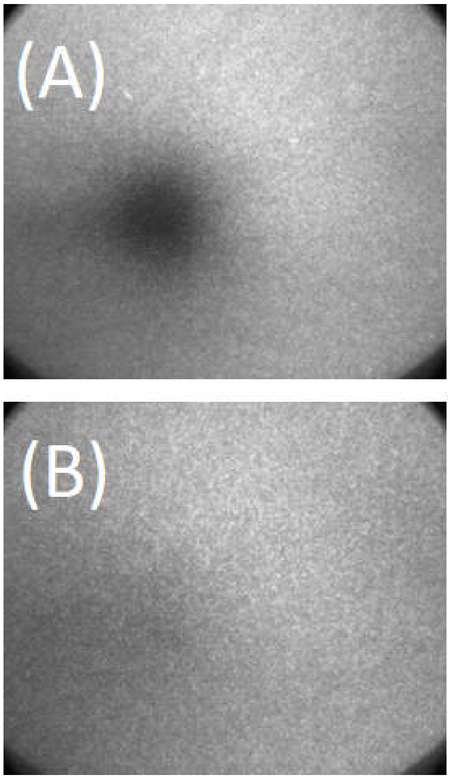

Figure S2. TIRF micrographs of a semi-native SLB highlighting (**A**) a photobleached hole, and (**B**) a recovered hole, demonstrating fluorescent recovery and mobility of labelled lipids within snSLB.
